# Impact of HIV-1 Subtype on the Time to CD4+ T-Cell Recovery in Combination Antiretroviral Therapy (cART)-Experienced Patients

**DOI:** 10.1371/journal.pone.0137281

**Published:** 2015-09-03

**Authors:** Wei Zhen Chow, Sin How Lim, Lai Yee Ong, Yean Kong Yong, Yutaka Takebe, Adeeba Kamarulzaman, Kok Keng Tee

**Affiliations:** 1 Centre of Excellence for Research in AIDS (CERiA), Department of Medicine, Faculty of Medicine, University of Malaya, Kuala Lumpur, Malaysia; 2 AIDS Research Center, National Institute of Infectious Diseases, Toyama, Shinjuku-ku, Tokyo, Japan; Fudan University, CHINA

## Abstract

Human immunodeficiency virus type 1 (HIV-1) subtypes have been shown to differ in the rate of clinical progression. We studied the association between HIV-1 subtypes and the rate of CD4+ T-cell recovery in a longitudinal cohort of patients on combination antiretroviral therapy (cART). We studied 103 patients infected with CRF01_AE (69%) and subtype B (31%) who initiated cART between 2006 and 2013. Demographic data, CD4+ T-cell counts and HIV-1 viral load were abstracted from patient medical charts. Kaplan-Meier was used to estimate the time to CD4+ T-cell count increase to ≥350 between subtypes and effects of covariates were analysed using Cox proportional hazards. An 87% of the study population were male adults (mean age of 38.7 years old). Baseline CD4+ T-cell counts and viral loads, age at cART initiation, sex, ethnicity and co-infection did not differ significantly between subtypes. A shorter median time for CD4+ T-cell count increase to ≥350 cells/μL was observed for CRF01_AE (546 days; 95% confidence interval [CI], 186–906 days; *P* = .502) compared to subtype B (987 days; 95% CI, 894–1079 days). In multivariate analysis, female sex was significantly associated with a 2.7 times higher chance of achieving CD4+ T-cell recovery (adjusted hazard ratio [HR], 2.75; 95% CI, 1.21–6.22; *P* = .025) and both baseline CD4+ T-cell count (*P* = .001) and viral load (*P* = .001) were important predictors for CD4+ T-cell recovery. Immunological recovery correlated significantly with female sex, baseline CD4+ T-cell counts and viral load but not subtype.

## Introduction

The HIV-1 epidemic involves the co-circulation of genetically-diverse subtypes of group M, comprising of 11 subtypes or sub-subtypes and 72 circulating recombinant forms (CRF). In Southeast Asia, it is estimated that approximately 1.8 million people live with HIV/AIDS (PLHIV) and CRF01_AE is the major circulating subtype (comprising approximately 80% of the total HIV-1 infections) besides subtype B [1]. Both genetically distinct lineages have been circulating in the region since their first description in the 1980s, and have been targeted in vaccine trials including the recent RV144 trial [2].

Various forms of HIV-1 subtypes have been shown to differ in the rate of disease progression [3]. Several studies have reported that individuals infected with subtype D have a more rapid progression to AIDS and AIDS-related death compared to subtype A, C and recombinants in Africa [4]. Similar findings were also reported in the United Kingdom where individuals infected with subtype D experienced faster rate of CD4+ T-cell decline resulting in a higher rate of virological failure compared to subtypes A, B, C and CRF02_AG [5]. In a cohort of female commercial sex workers in West Africa, the rate of disease progression to AIDS was eight-fold higher in the study population infected with non-A subtypes (C, D and G) compared to subtype A [6]. White individuals infected with subtype B experienced higher rates of virological failure following the initiation of combination antiretroviral therapy (cART) compared to non-B subtypes (CRF02_AG, CRF01_AE, A and C) [7].

In Southeast Asia, previous studies have described the association between the major circulating subtypes—CRF01_AE and subtype B and the rate of disease progression, particularly among HIV-1 seroconverters [8]. Patients infected with CRF01_AE subtype experienced a significantly faster rate of CD4+ T-cell decline and hence initiated cART earlier compared to non-CRF01_AE (subtype B, CRF33_01B, CRF34_01B and G) [8]. At present, little is known about the role of HIV-1 subtype in determining the response to therapy, as defined by the rate of CD4+ T-cell recovery during cART. It is therefore crucial to investigate whether subtype may predict the rate of CD4+ T-cell recovery in patients initiating cART. The present study examined the rate of CD4+ T-cell recovery in a retrospective patient cohort initiating cART and infected with CRF01_AE or subtype B in Kuala Lumpur, Malaysia.

## Methods

The study was approved by the UMMC Medical Ethics Committee and all methods were carried out in accordance with approved guidelines. Written informed consent was obtained from patients prior to enrolment in the study.

### Study Population

By medical chart review, we abstracted demographic and clinical data of 103 patients who presented with chronic HIV-1 infection and attended the University Malaya Medical Centre (UMMC) in Kuala Lumpur, Malaysia between 2006 and 2013. Eligible individuals were ARV-naïve patients who initiated first-line cART (consisting of stavudine, lamivudine and efavirenz), self-reported adherence to therapy and attended subsequent follow-ups at the infectious diseases clinic from the time of HIV-1 diagnosis or cART initiation. We excluded five individuals who were infected with minor subtype [CRF33_01B and other unique recombinant forms (URF)] due to inadequate number of cases. Important clinical data collected included baseline and serial assessments of CD4+ T-cell counts (every six months) and plasma HIV-1 RNA viral load (once or twice yearly), follow-up duration, history of cART prescription and co-infection with either tuberculosis or hepatitis B/C (HBV/HCV). Basic patient demographic information included age at the initiation of cART, sex, ethnicity and route of disease transmission. Baseline HIV-1 genotyping was performed using our in-house nested PCR amplification and population sequencing of the *gag*-RT gene (HXB2: 1753–3440 nt), followed by standard phylogenetic tree analyses [9]. All laboratory tests were performed at the UMMC. CD4+ T-cells were enumerated using the BD FACSCalibur platform (BD Biosciences, USA) and plasma HIV-1 RNA levels were measured using the COBAS TaqMan HIV-1 assay (Roche, Switzerland), with a lower limit of detection of <40 copies/mL, according to manufacturer’s protocol. Baseline HIV-1 genotyping test was conducted, CD4+ T-cell counts and plasma HIV-1 RNA measurements were taken prior to initiation of cART. We analyzed the immunological response to cART by measuring the rate of CD4+ T-cell recovery, as defined by the time to achieve CD4+ T-cell count to ≥350 or ≥500 cells/μL [10].

### Statistical Analysis

Patients’ baseline clinical and demographic characteristics were compared between HIV-1 subtype groups using an independent t-test and Mann-Whitney U test as appropriate (*P* < .05 is considered significant). Kaplan-Meier and log-rank test were used to estimate the time to CD4+ T-cell count increase to ≥350 cells/μL upon cART initiation between the infecting subtypes. Effects of covariates were analysed using Cox proportional hazards and important covariates (*P* < .25 is considered statistically significant) were included in the subsequent multivariate analysis [11]. All analyses were performed on SPSS software version 21 (IBM).

## Results

### Baseline characteristics of the cohort

A total of 103 HIV-1-infected patients who initiated cART was analysed. A majority of patients was men (87.4%), of Chinese ethnicity (75.7%), and with a mean age of 38.7 years old (ranging from 30.7–46.7 years old). The basic demographic and relevant clinical data were summarized in Table 1. About 69% of the patients were infected with CRF01_AE and 31% with subtype B. Approximately 63% of patients acquired HIV-1 infection through heterosexual activity and 30% included men who have sex with men (MSM) or people who inject drugs (PWID). The rate of co-infection in this population was relatively low, with 17.5% diagnosed with either tuberculosis or hepatitis B/C. The median period of follow up is 1005.0 days (IQR, 568.5–1395.5 days). Patients were censored at the time point when they were lost to follow up or when treatment was interrupted. Allergic reactions, depression, and financial constraints were some of the reasons given for patients being lost to follow up. Throughout the study period, all participants did not acquire drug resistance mutation while on therapy, confirmed by routine drug resistance test. Overall baseline CD4+ T-cell counts and log-transformed viral loads, age at cART initiation, sex, ethnicity and co-infection did not differ significantly across subtypes, except for the route of disease transmission (P = .004).

**Table 1:** Basic demographic and baseline clinical characteristics of 103 HIV-1 patients initiating cART in Kuala Lumpur, Malaysia.

	HIV-1 subtype		
	CRF01_AE	B	
Characteristics	71 (68.9)	32 (31.1)	*P* value ^ψ^
**Sex**			0.195^€^
Male	60 (84.5)	30 (93.8)	
Female	11 (15.5)	2 (6.3)	
**Ethnicity**			0.110^€^
Chinese	57 (80.3)	21 (65.6)	
Non-Chinese^a^	14 (19.7)	11 (34.4)	
**Route of transmission**			0.004^€^
Heterosexual	50 (70.4)	15 (46.9)	
Non-heterosexual^b^	15 (21.1)	16 (50.0)	
Unknown	6 (8.5)	1 (3.1)	
**Clinical parameters**			
Age at starting cART, years, mean (± SD)	38.7 (± 8.9)	38.2 (± 8.3)	0.762^€^
**Baseline CD4+ T cell count, cells/μL, median (IQR)**	64.0 (16.5–240.5)	84.0 (29.8–209.8)	0.608^**Δ**^
≤200	49 (69.0)	24 (75.0)	
201–249	5 (7.0)	1 (3.1)	
250–299	9 (12.7)	4 (12.5)	
300–349	8 (11.3)	3 (9.4)	
Baseline HIV-1 RNA, log copies/mL, mean (± SD)	5.07 (± 0.886)	4.88 (± 0.533)	0.253^€^
Time from presentation to cART initiation, days	34.0 (14.0–120.5)	17.5 (7.8–60.5)	0.081^**Δ**^
Follow-up duration, days^c^	1005.0 (568.5–1395.5)	1298.5 (564.3–1567.0)	0.120^**Δ**^
**Co-infection** ^d^			0.057^**Δ**^
Yes	9 (12.7)	9 (28.1)	
No	62 (87.3)	23 (71.9)	

Categorical variables are described as n (%) and numerical variables are described as mean (± SD) or median (IQR).

Abbreviations: HIV-1, human immunodeficiency type-1; cART, combination antiretroviral therapy; SD, standard deviation; IQR, interquartile range.

^ψ^ *P* values are given for test of general differences across the two subgroups using an independent t-test^€^ for normally-distributed categorical outcomes and Mann-Whitney U test^Δ^ for non-normally-distributed continuous outcomes (*P* values of < .05 are considered significant).

^a^ data includes Malay, Indian and other minor ethnicities.

^b^ data includes men-who-have-sex-with-men (MSM), unsafe injecting drug use and blood transfusion.

^c^ the interval between the day of cART initiation and the last clinical consultation available.

^d^ includes co-infections with tuberculosis, hepatitis B or C virus (HBV/HCV).

### Association between HIV-1 subtypes and the rate of CD4+ T-cell recovery

Approximately 70.9% of patients had a baseline CD4+ T-cell count of ≤200 cells/μL prior to cART initiation. At the end of the study follow-up period (median, 33.5 months; IQR, 19.0–46.5 months), 59.2% achieved a CD4+ T-cell recovery to ≥350 cells/μL and within this group, 57.4% (n = 35/61) achieved a CD4+ T-cell count of ≥500 cells/μL and the remaining 42.6% did not achieve CD4+ T-cell count of ≥500 cells/μL. On the other hand, 40.8% (n = 42/103) of the population did not achieve CD4+ T-cell count of ≥350 cells/μL at the end of follow up.

**Fig 1:**
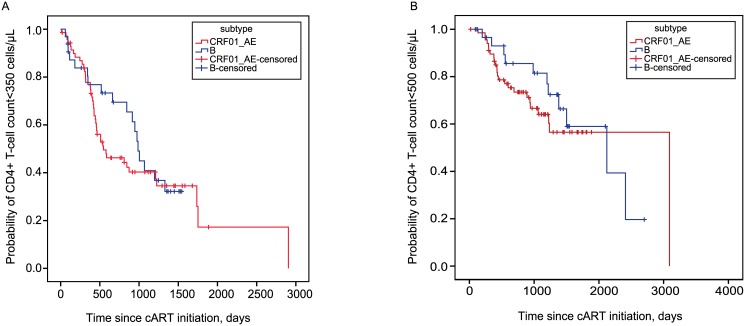
Kaplan-Meier analysis of time to reach CD4+ T-cell count of ≥350 cells/μL (*A*) or to ≥500 cells/μL (*B*) in patients infected with CRF01_AE and subtype B on cART. *P* values were calculated using log-rank test.

In the Kaplan-Meier analysis, a shorter median time to CD4+ T-cell count increase ≥350 cells/μL was observed in CRF01_AE-infected population (546.0 days; 95% confidence interval [CI], 186.0–906.0 days; *P* = .502) compared to subtype B (987.0 days; 95% CI, 894.9–1079.1 days) (Fig 1A). The current (WHO) treatment guidelines recommend healthcare providers to initiate cART when the CD4+ T-cell count falls <500 cells/μL, therefore we analysed the time taken for CD4+ T-cell recovery to ≥500 cells/μL in the sub-analysis. The Kaplan-Meier analysis showed that subtype B-infected population was associated with a shorter median time to CD4+ T-cell count increase to ≥500 cells/μL (2122.0 days; 95% confidence interval [CI], 1023.6–3220.4 days; *P* = .458) compared to CRF01_AE (3090.0 days) (Fig 1B). However, the difference observed in the time taken to achieve a CD4+ T-cell count to either ≥350 or ≥500 cells/μL between subtype B and CRF01_AE-infected patients was not statistically significant.

Next, we evaluated the effects of other covariates on HIV-1 disease progression and response to treatment (Table 2). In the univariate analyses, female sex, non-Chinese ethnicity age and timing of cART initiation and, both baseline CD4+ T-cell count (*P* = .001 and log-transformed HIV-1 RNA viral load (*P* = .257) and absence of co-infections (*P* = .050) were significantly associated with the rate of CD4+ T-cell recovery to ≥350 cells/μL. In the multivariate analyses, female sex is significantly associated with a faster rate of CD4+ T-cell recovery to ≥350 cells/μL (adjusted HR; 2.75; 95% CI, 1.21–6.22; *P* = .025) compared to the males. Both baseline CD4+ T-cell count (adjusted HR; 1.008; 95% CI, 1.005–1.011; *P* < .001) and log-transformed HIV-1 RNA viral load (adjusted HR; 2.035; 95% CI, 1.273–3.252; *P* < .001) and the follow-up period (*P* < .001) were strongly associated with the rate of CD4+ T-cell recovery in this population. In the adjusted analyses, non-Chinese ethnicity (*P* = .173), age (*P* = .448) and timing of cART initiation (*P* = .060) and co-infections (*P* = .067) were not significantly associated with the rate of CD4+ T-cell recovery.

**Table 2:** Cox proportional hazards using unadjusted (univariate) and adjusted (multivariate) analyses to assess the effects of covariates on the rate of CD4+ T cell recovery, defined as time for CD4+ T-cell count increase to ≥350 cells/μL during cART.

	Unadjusted analysis		Adjusted analysis^a^	
Covariates	HR (95% CI)	*P* value	HR (95% CI)	*P* value
**Sex**				
Male	**1.0 (Ref)**		**1.0 (Ref)**	
Female	1.594 (.777–3.267)	.203	2.748 (1.214–6.222)	.025
**Ethnicity**				
Chinese	**1.0 (Ref)**		**1.0 (Ref)**	
Non-Chinese	1.502 (.862–2.619)	.151	1.509 (.835–2.727)	.173
**Routes of transmission**				
Heterosexual	**1.0 (Ref)**		**1.0 (Ref)**	
Non-heterosexual	0.732 (.256–2.092)	.561		
Unknown	0.880 (.296–2.620)	.819		
**Clinical parameters**				
Age at starting cART, years	.975 (.946–1.006)	.109	.988 (.957–1.019)	.448
Baseline CD4+ T cell count, cells/μL	1.007 (1.005–1.010)	< .001	1.008 (1.005–1.011)	< .001
Baseline HIV-1 RNA, log copies/mL	1.263 (.843–1.893)	.257	2.035 (1.273–3.252)	.001
Time from presentation to cART initiation, days	1.000 (1.000–1.001)	.011	1.001 (1.000–1.001)	.060
Follow-up duration, days	0.999 (.998–1.000)	< .001	0.999 (.998–1.000)	< .001
**Co-infection**				
Yes	**1.0 (Ref)**		**1.0 (Ref)**	
No	.429 (.184–1.000)	.050	0.477 (.202–1.131)	.067

Abbreviations: cART, combination antiretroviral therapy; HR, hazard ratio; CI, confidence intervals; Ref, reference.

^a^ covariates with P values < .25 were included in the adjusted analysis. The rate of CD4+ T cell recovery was also adjusted for baseline HIV-1 RNA because it is known to be an important predictor of immunologic recovery.

## Discussion

Since the advent of cART in the 1990s, a longer life expectancy and better prognosis are now attainable among 12.9 million people living with HIV worldwide. However, the number of individuals who had access to therapy is a staggering proportion of the total 35 million PLHIV within that year (UNAIDS Fact Sheet 2014). In Malaysia, nearly half, that is 17,369 individuals of the total 38,420 PLHIV received cART in 2013 (Global AIDS response progress report Malaysia 2014, Ministry of Health). In the present study, we studied the immunological response in HIV-1 patients infected with different subtypes in Kuala Lumpur, Malaysia by measuring the time from initiating cART to CD4+ T-cell recovery to ≥350 or ≥500 cells/μL within a median follow-up period of 2.8 years. We have shown that the rate of CD4+ T-cell recovery to either ≥350 or ≥500 cells/μL did not differ significantly between patients infected with either subtype B or CRF01_AE, hence suggesting that immunological response in our study population was relatively independent of the infecting subtypes. A recent systematic review on published findings on the impact of non-B subtypes towards disease progression in patients on cART corroborated our findings, in which it was concluded that the difference in disease progression was minimalized in cART-experienced patients [12]. It remains uncertain as to why subtype was not an important predictor of CD4+ T-cell recovery in cART-experienced patients in our study, however we speculate that further studies on characterising the viral latent reservoir in these patients may provide a clue on the possible viral mechanism or host factors involved as recent evidence supports a linkage between the latent reservoir and disease progression. The levels of total and integrated HIV-1 DNA have recently been reported as one of the strong predictors of disease progression in patients with primary HIV-1 infection [13].

In a study examining the association between the main co-circulating subtypes in Singapore and disease progression, HIV-1 seroconverters were followed from the estimated date of seroconversion to the time when cART was initiated [8]. CRF01_AE-infected seroconverters were found to experience a faster rate of CD4+ T-cell decline and thus required an earlier cART initiation compared to non-CRF01_AE [8]. In another study, Ng et al. also reported that non-B-infected patients had lower CD4+ T-cell counts at 9–15 months after cART initiation than subtype B-infected patients. However, the difference observed may be related to a significantly higher baseline CD4+ T-cell counts in the subtype B-infected patients compared to non-B subtypes prior to cART initiation [14]. Little is known about whether the subtype-associated differences observed pre-cART may exist in treatment-experienced patients and studies addressing this aspect were limited to mostly seroconverters. Findings from our study suggest that subtype B and CRF01_AE do not affect the rate of CD4+ T-cell recovery in patients initiating cART. The rate of immunological recovery was associated with female sex, baseline CD4+ T-cell count and baseline HIV-1 viral load in our cohort. However, since our study population mainly presented late in the course of HIV-1 infection, with approximately 71% of patients having a CD4+ T-cell count ≤200 cells/μL, we were unable to directly compare the subtype-associated differences before and after cART initiation within the same population and this aspect certainly warrants future investigation. In addition, the findings may be limited by the moderate sample size and thus may not be generalizable to other populations with different level of host immune activation [15] and human leukocyte antigen (HLA) genotypes [16]. Nevertheless, the moderate sample size of our study was comparable to the previous studies analysing the association between HIV-1 subtypes or dual HIV-1 infections and disease progression with sample size between six and 70 patients per subtype group [8,14,17]. These studies contributed significantly to the understanding of viral factors which affect disease progression in HIV-1 infected patients in different cohorts despite the moderate sample size.

Similar to previous studies, both baseline CD4+ T-cell counts [18] and plasma HIV-1 viral load [19] are strong predictors of disease progression in HIV-1-infected patients. Indeed the presence of baseline HIV-1 specific CD4+ responses in PHI was strongly correlated with slower disease progression in the recent SPARTAC trial [20]. In the present study both baseline CD4+ T-cell counts and plasma HIV-1 viral load were important predictors of CD4+ T-cell recovery over a median follow-up period of 2.8 years. Given that both baseline CD4+ T-cell counts and log-transformed plasma HIV-1 viral load did not differ significantly between CRF01_AE and subtype B-infected patients, our conclusions are less likely to be biased towards favouring the rate of CD4+ T-cell recovery in either subtype. The relationship between male/female and HIV-1 disease progression has been investigated for decades and sex differences significantly impacts the clinical, virological and immunological responses in patients initiating cART [21]. In an earlier study by Farzadegan et al., female HIV-1 seroconverters had up to 40% lower HIV-1 plasma viral load compared to men although at similar viral load, women were found to have a 1.6-fold higher risk of progressing towards AIDS [22]. Laboratory research suggests that the production of interferon-α (IFN-α) by the plasmacytoid dendritic cells (pDCs) and the upregulation of interferon-stimulated genes (ISG) were significantly higher in women compared to HIV-1-infected men [23,24]. These factors may contribute to a higher immune activation and hence faster disease progression in chronically-infected HIV-1 women [25]. Nicastri et al. reported that sex-based differences were not observed in terms of virological suppression or failure among the patients on cART, however at the baseline HIV-1 viral load of 10^4^–10^5^ copies/mL, HIV-1-infected women had a better clinical outcome compared to men [26]. In another study by Collazos et al., HIV-1-infected women who were receiving cART had better immunological response to therapy and hence higher CD4+ T-cell counts and lower rate of clinical progression to AIDS/death compared to men [21]. In this study, we also observed that HIV-1-infected women had at least 2.7 times significantly higher chance of achieving CD4+ T-cell count of ≥350 cells/μL compared to the HIV-1-infected men during cART, implying that the immunological response to therapy was better in women. However it is unknown whether sex-based differences will affect the rate of disease progression in our current study population which is the time to AIDS/death and this certainly warrants future investigation in a larger cohort and with a longer follow-up period.

The differential rates of disease progression in HIV-1-infected persons is complex as it involves an interplay of the host and viral factors in the population. Our present study found that CRF01_AE and subtype B were not important predictors of CD4+ T-cell recovery in a cART-experienced population. On the other hand, female sex and both baseline CD4+ T-cell counts and HIV-1 viral load were strong predictors of CD4+ T-cell recovery during cART. Therefore, an earlier cART initiation as recommended by the 2013 WHO treatment guidelines for CD4+ T-cell count <500 cells/μL may prove to be useful in promoting CD4+ T-cell recovery in patients receiving cART. Furthermore, current treatment strategies and prevention efforts should take into account the sex-based differences in immunological response to therapy in order to greatly reduce HIV-1/AIDS-associated morbidity and mortality.
